# Intracellular Vesicle Trafficking Genes, *RabC*-GTP, Are Highly Expressed Under Salinity and Rapid Dehydration but Down-Regulated by Drought in Leaves of Chickpea (*Cicer arietinum* L.)

**DOI:** 10.3389/fgene.2019.00040

**Published:** 2019-02-07

**Authors:** Gulmira Khassanova, Akhylbek Kurishbayev, Satyvaldy Jatayev, Askar Zhubatkanov, Aybek Zhumalin, Arysgul Turbekova, Bekzak Amantaev, Sergiy Lopato, Carly Schramm, Colin Jenkins, Kathleen Soole, Peter Langridge, Yuri Shavrukov

**Affiliations:** ^1^Faculty of Agronomy, S. Seifullin Kazakh AgroTechnical University, Astana, Kazakhstan; ^2^Biological Sciences, College of Science and Engineering, Flinders University, Bedford Park, SA, Australia; ^3^School of Agriculture, Food and Wine, University of Adelaide, Adelaide, SA, Australia; ^4^Wheat Initiative, Julius-Kühn-Institute, Berlin, Germany

**Keywords:** abiotic stresses, Amplifluor-like SNP markers, bioinformatics, *CaRab* gene, differential gene expression, gene isoforms

## Abstract

Intracellular vesicle trafficking genes, *Rab*, encoding small GTP binding proteins, have been well studied in medical research, but there is little information concerning these proteins in plants. Some sub-families of the *Rab* genes have not yet been characterized in plants, such as *RabC* – otherwise known as *Rab18* in yeast and animals. Our study aimed to identify all *CaRab* gene sequences in chickpea (*Cicer arietinum* L.) using bioinformatics approaches, with a particular focus on the *CaRabC* gene sub-family since it featured in an SNP database. Five isoforms of the *CaRabC* gene were identified and studied: *CaRabC-1a, -1b, -1c, -2a* and *-2a^∗^*. Six accessions of both Desi and Kabuli ecotypes, selected from field trials, were tested for tolerance to abiotic stresses, including salinity, drought and rapid dehydration and compared to plant growth under control conditions. Expression analysis of total and individual *CaRabC* isoforms in leaves of control plants revealed a very high level of expression, with the greatest contribution made by *CaRabC-1c*. Salinity stress (150 mM NaCl, 12 days in soil) caused a 2-3-fold increased expression of total *CaRabC* compared to controls, with the highest expression in isoforms *CaRabC-1c, -2a^∗^* and *-1a*. Significantly decreased expression of all five isoforms of *CaRabC* was observed under drought (12 days withheld water) compared to controls. In contrast, both total *CaRabC* and the *CaRabC-1a* isoform showed very high expression (up-to eight-fold) in detached leaves over 6 h of dehydration. The results suggest that the *CaRabC* gene is involved in plant growth and response to abiotic stresses. It was highly expressed in leaves of non-stressed plants and was down-regulated after drought, but salinity and rapid dehydration caused up-regulation to high and very high levels, respectively. The isoforms of *CaRabC* were differentially expressed, with the highest levels recorded for *CaRabC-1c* in controls and under salinity stress, and for *CaRabC-1a* – in rapidly dehydrated leaves. Genotypic variation in *CaRabC-1a*, comprising eleven SNPs, was found through sequencing of the local chickpea cultivar Yubileiny and germplasm ICC7255 in comparison to the two fully sequenced reference accessions, ICC4958 and Frontier. Amplifluor-like markers based on one of the identified SNPs in *CaRabC-1a* were designed and successfully used for genotyping chickpea germplasm.

## Introduction

Plant genomes include a superfamily of genes that encode small GTP-binding proteins (Guanosine triphosphatases) that are classified into four groups: *Arf, Rab, Ran* and *Rho*; and an additional *Ras*-GTP gene group is found only in yeast and animals (Ma, 2007). Small GTP-binding proteins were first described in medical research, where the term “*Ras*” stemmed from their association with rat sarcoma (Chang et al., 1982; Bishop, 1985; Chavrier et al., 1990). The remaining three-letter names are not related in structure or function to the genes but rather refer to their product or some other feature (Coffin et al., 1981). Small GTP-binding proteins are known to be involved in a diverse range of activities in eukaryotes that are vital for growth, development and repair; from cytoskeletal organization, vacuolar storage and signaling, to modulation of gene expression (Takai et al., 2001). The mechanism for the regulation of GTP-binding proteins is conserved in all organisms and involves cycling between active (GTP-bound) and inactive (GDP-bound) forms, so they are often described as “molecular switches” that are turned “on” or “off” via the hydrolysis of GTP (Marshall, 1993). Activation requires the dissociation of GDP, which can be either stimulated by a regulatory factor named GEP (GDP/GTP Exchange Protein) or inhibited by GDI (GDP Dissociation Inhibitor; Takai et al., 2001; Liu et al., 2015; Martín-Davison et al., 2017).

Rab proteins, encoded by *Rab*-GTP genes, are normally prenylated at their carboxyl terminus. The hydrophobic prenyl-groups facilitate attachment to membranes and are therefore integral to the biological role performed by Rab proteins in vesicle trafficking via endocytic and exocytic pathways between the endoplasmic reticulum, Golgi membrane network, endosome, plasma membrane and all intracellular membranes (Alory and Balch, 2003). Rab proteins are highly conserved across kingdoms, from yeast to animals and plants (Haubruck et al., 1987; Marcote et al., 2000), but are most often present as a small family of highly similar genes. They are divided into either nine (Ma, 2007) or 18 clades (Agarwal et al., 2009) based on their structure, with only eight clades represented in plants. Historically, different nomenclatures were adopted for identification of *Rab* genes in plants compared to animals. For example, in plants, eight capitalized letters from A to H were used in the names of *Rab* genes, while the numbers 1 to 11 were applied in human, animal and yeast research. In the absence of a universal system of nomenclature for *Rab* genes and their proteins, a list of all known genes and their respective identifiers for both nomenclatures is given later in the text.

The genes for Rab GTP-binding proteins should not be confused with the similarly named *Dehydrin* genes in plants, which are also known as *RAB*, meaning “Responsive to ABA” (Abscisic acid). *Dehydrins* encode proteins belonging to the large but very different group of Late embryogenesis abundant proteins, LEA (Hundertmark and Hincha, 2008). For example, *AtRAB18* (or *AtRab18*) was described and studied in *Arabidopsis thaliana* in response to various abiotic stresses and ABA treatment (Lång and Palva, 1992; Rushton et al., 2012; Hernández-Sánchez et al., 2017). Despite the identical name, this gene is neither structurally nor functionally related to the *Rab-*GTP genes, and care must be taken to clearly distinguish between the two. The mixing of these two different types of genes is unfortunately apparent in recent publications. For example, Jiang et al. (2017) studied the correctly designated *TaRab18* (=*TaRabC1*) gene in response to stripe rust in bread wheat, but this gene was incorrectly compared with *RAB18* (Responsive to ABA) in *Arabidopsis*, rice and maize. As a result, the Authors wrongly cited work by Lång and Palva (1992) and others on the *Dehydrin AtRab18* to support their findings on the sensitivity of *TaRab18* (=*TaRabC1*) to ABA.

In plants, *Rab-*GTP genes are reportedly involved in multiple physiological processes (Borg et al., 1997; Rehman and Sansebastiano, 2014; He et al., 2018; Lawson et al., 2018) and are often highly expressed in response to biotic and abiotic stresses (Marcote et al., 2000; Stenmark and Olkkonen, 2001; Zerial and McBride, 2001; Rutherford and Moore, 2002; Ma, 2007; Woollard and Moore, 2008; Agarwal et al., 2009). However, despite the numerous links, little is known about the precise molecular mechanisms underlying their involvement in plant stress responses.

One of the first studies to report a link between Rab protein and abiotic stress was a report by O’Mahony and Oliver (1999) who found increased transcript levels of the *Rab2* gene (otherwise known as *RabB*) in the desiccation-tolerant grass *Sporobolus stapfianus* in response to dehydration, but decreased transcript levels after rehydration. This suggested the involvement of *SsRab2* in both the short-term response and later recovery from desiccation. SsRab2 was found to share 90% similarity to Rab2 proteins found in rice, maize, *Arabidopsis, Lotus japonicus* and soybean (O’Mahony and Oliver, 1999). Since that time, links to various stresses have been established for genes encoding Rab proteins in numerous plants, and especially in species with high abiotic stress-tolerance such as *Lilium formolongi* – *LfRabB* (Howlader et al., 2017), poplar – *PtRabE1b* (Zhang et al., 2018), and *Mesembryanthemum crystallinum* – *McRab5b* (=*McRabF*) (Bolte et al., 2000). Interestingly, many plant species were studied for *RabG* genes and their corresponding proteins including the halophyte species, *Aeluropus lagopoides* – *AlRab7* (=*AlRabG*) (Rajan et al., 2015) and food grain crop, *Pennisetum glaucum* – *PgRab7* (=*PgRabG*) (Agarwal et al., 2008), as well as more stress susceptible crops such as rice, *Oryza sativa* – *OsRab7* (=*OsRabG*) (Nahm et al., 2003) and peanut, *Arachis hypogaea* – *AhRabG* (Sui et al., 2017), and the model species *A. thaliana* – *AtRab7* (=*AtRabG*) (Mazel et al., 2004). A comprehensive analysis of all *MpRab* genes was reported for the liverwort, *Marchantia polymorpha* (Minamino et al., 2018).

Rab transcripts are often found to show different responses to abiotic stresses. For example, in rice, dehydration triggered a strong increase in *OsRab7* (=*OsRabG*) transcript after 4 h and then a decrease after 10 h. However, no significant changes were found in response to cold or salinity stress (Nahm et al., 2003). Similarly, in the halophytic grass *A. lagopoides, AlRab7* (=*AlRabG*) was upregulated by dehydration, but salinity stress caused no significant increase in transcript levels (Rajan et al., 2015). In another halophyte, *M. crystallinum*, expression of *McRab5b* (=*McRabF*) was higher after 2 h and continued to rise over 3 days of very strong salt stress (400 mM NaCl), but wilting or osmotic stress triggered no change in expression (Bolte et al., 2000). These differences obviously reflect various roles of the intracellular membrane system to abiotic stresses and may provide the key to uncovering the precise molecular mechanisms underlying differential plant susceptibility or tolerance to an environmental stress.

A number of studies have used a transgenic approach to shed light on the mechanisms explaining the link between Rab proteins and plant stress and to explore how Rab proteins could play a role in the breeding of more stress-tolerant crops. For example, Mazel et al. (2004) constitutively overexpressed *AtRabG3e* (=*AtRab7*) in *Arabidopsis*. The transgenic plants accumulated more sodium in vacuoles and showed greater tolerance to salinity and osmotic stress. Evidence was also found for increased endocytosis in roots and leaves and entry of Reactive oxygen species into the cell to trigger signaling and subsequent activation of stress tolerance mechanisms (Mazel et al., 2004; Baral et al., 2015). *AhRabG, OsRab7 (=OsRabG)* and *OsRab11* (=*OsRabA*) were also overexpressed in transgenic peanut and rice, respectively, producing plants that showed relatively higher salinity tolerance compared to wild-type plants (Peng et al., 2014; Sui et al., 2017; Chen and Heo, 2018). In transgenic peanut plants, of 132 genes differentially expressed, most were identified as transcription factors (TF) relating to salinity tolerance (Sui et al., 2017).

The aim of this study was to identify and analyze a possible candidate gene involved in the tolerance to drought, salinity and rapid dehydration in chickpea, *C. arietinum*, a species that is becoming increasingly popular as a cash crop in agricultural areas with the requirements for moderate tolerance to high temperatures, drought and salinity stress during the growing season. A candidate gene *CaRabC1*, belonging to the family of *Rab-*GTP genes, was identified from an SNP database using bioinformatic and molecular genetic analyses. Currently, the only report concerning chickpea *Rab-*GTP genes was published by Muñoz et al. (2001), who identified a Rab-specific GDI in chickpea seedlings showing 96% homology to *MsRab11f* (=*MsRabG*), a GDI in *Medicago truncatula* (Yaneva and Niehaus, 2005). Our study therefore represents the first report of the *Rab-*GTP family of genes in *C. arietinum.* We present the results of bioinformatic analyses of the identified genes and tests conducted to assess the expression of all isoforms of the *CaRabC* gene family in response to salinity, drought and rapid dehydration in selected chickpea genotypes. Amplifluor-like markers based on one of the identified SNPs in *CaRabC-1a* were used for genotyping of chickpea germplasm.

## Materials and Methods

### Plant Material

A germplasm collection comprising 250 chickpea (*C. arietinum* L) samples from the ICRISAT Reference set plus local accessions were tested over 3 years in field trials in Northern and Central Kazakhstan. Six accessions were selected during field trials for further molecular analyses, as listed in Table 1. The first accession, cv. Yubileiny, originated from Krasnokutskaya Breeding Station, in the Saratov region (Russia), and is used as a Standard for local field trials with chickpea accessions. The remaining five chickpea lines were selected from the original 230 collected in the ICRISAT Reference set, to represent diverse gene-pool sources.

**Table 1:** List and short description of six selected chickpea germplasm accessions used for molecular analyses.

Code	Name	Cultivar/Landrace/Line	Ecotype	Origin
Yub	Yubileiny	Cultivar	Kabuli	Russia
ICC-4841	P6615	Landrace	Kabuli	Morocco
ICC-7255	NEC1628; SN8	Landrace	Kabuli	India
ICC-1392	P1240; 141-1	Landrace	Desi	India
ICC-4918		Elite line	Desi	India
ICC-12726	RFA100-3	Landrace	Desi	Ethiopia

### Identification of the Gene of Interest Using Bioinformatics and Molecular Phylogenetic Comparative Analysis

Bioinformatics and systems biology methods were applied in this study to identify a target gene or “Gene of Interest” (GoI) with a potential role in tolerance to abiotic stresses in chickpea. Initially, the SNP database for *C. arietinum* L.^1^ was used to search and select one suitable SNP with a short fragment of sequence for further study. The full-length nucleotide sequence of the GoI and its corresponding polypeptide sequence was retrieved from the same database and used for both BLASTN and BLASTP in NCBI and in GenomeNet Database Resources, hosted by Kyoto University, Japan^2^. All chickpea gene sequences with KEGG and NCBI identification and the encoded proteins were downloaded from GenomeNet and NCBI databases, while chromosome locations were checked using LIS, Legume Information System database^3^. The *A. thaliana* genes displaying the highest level of similarity to each GoI within the gene family were identified using alignments from the same database.

Multiple sequence alignments of nucleotide sequences for the *Rab* family of genes were conducted in CLUSTALW using GenomeNet Database Resources^4^, while CLC Main Workbench software^5^ was used for protein amino acid sequence alignment.

The molecular dendrogram was constructed using BLASTP at GenomeNet Database Resources (See footnote 2) with the function of ETE3 v3.0.0b32 (Huerta-Cepas et al., 2016) and MAFFT v6.861b applied using the default options (Katoh and Standley, 2013). The FastTree v2.1.8 program with default parameters was used for phylogenetic tree preparation (Price et al., 2009).

### Abiotic Stress Treatments: Salinity, Drought and Rapid Dehydration

Three experiments applying abiotic stress treatments (salinity, slow drought and rapid dehydration) were carried out in chickpea for RT-qPCR analyses using the same conditions as described earlier in our publication for wheat (Zotova et al., 2018). The size of containers used, number of plants, soil type and growth conditions were all as described and no artificial inoculation of rhizobium was applied.

For salt stress, twenty-four uniform seedlings in each of six accessions were grown for one month in two separate containers. On “Day 0,” three plants from each accession (three biological replicates) were randomly selected from each container, before the salt stress was applied. The two youngest fully developed leaves were collected from each selected plant into a 10-ml plastic tube and immediately frozen in liquid nitrogen and stored at –80°C until RNA extraction. Subsequently, 200 ml of 150 mM NaCl was applied to the container, covering the entire soil surface but avoiding any direct contact with the plants. The NaCl treatments were applied four times, on every third day following Day 0 (over 12 days in total) in treatment containers, while the same volume of tap-water without NaCl was used under the same schedule in the control containers. No solution was lost through drainage from any container. No supplementary CaCl_2_ was added despite the recommended requirements in experiments with hydroponics. This is because the soil mix used contained sufficient available calcium and no symptoms of Ca deficiency were apparent in the treated plants. After 12 days, as on Day 0, the two youngest fully developed leaves were collected from each of three plants both in salt treatments and controls. Leaf samples were immediately frozen in liquid nitrogen and stored at –80°C for RNA extraction.

Experiments with slowly droughted plants and rapid dehydration of detached leaves were carried out using exactly the same schedule as described in Experiments 1 and 2 in our previous paper on wheat (Zotova et al., 2018).

### RNA Extraction, cDNA Synthesis and qPCR Analysis

Frozen leaf samples were ground as described below for DNA extraction. TRIzol-like reagent was used for RNA extraction following the protocol described by Shavrukov et al. (2013) and all other steps for RNA extraction and cDNA synthesis were as described previously (Zotova et al., 2018). The procedures included DNase treatment (Qiagen, Germany), and the use of a MoMLV Reverse Transcriptase kit (Biolabmix, Novosibirsk, Russia). All cDNA samples were checked for quality control using PCR and yielded bands of the expected size on agarose gels.

Diluted (1:2) cDNA samples were used for qPCR analyses using either a QuantStudio-7 Real-Time PCR instrument (Thermo Fisher Scientific, United States) at S. Seifullin Kazakh AgroTechnical University, Astana, Kazakhstan, or Real-Time qPCR system, Model CFX96 (BioRad, Gladesville, NSW, Australia) at Flinders University, Australia. The qPCR protocol was similar in both instruments as published earlier (Shavrukov et al., 2016), wherein the total volume of 10 μl q-PCR reactions included either 5 μl of 2xBiomaster HS-qPCR SYBR Blue (Biolabmix, Novosibirsk, Russia) for experiments in Kazakhstan or 5 μl of 2xKAPA SYBR FAST (KAPA Biosystems, United States) for experiments in Australia, 4 μl of diluted cDNA, and 1 μl of two gene-specific primers (3 μM of each primer) (Supplementary material 1). Expression data for the target genes were calculated relative to the average expression of the two reference genes: *CAC*, Clathrin adaptor complexes, medium subunit (Reddy et al., 2016) and *GAPDH*, Glyceraldehyde-3-phosphate dehydrogenase (Garg et al., 2010) (Supplementary material 1). At least three biological and two technical replicates were used in each qPCR experiment.

### DNA Extraction, Sequencing and SNP Identification

Plants were grown in control (non-stressed) conditions in containers with soil as described above. Five uniform one month-old individual plants were selected from each accession and five leaves were collected and bulked for leaf samples. Leaf samples frozen in liquid nitrogen were ground in 10-ml tubes with two 9-mm stainless ball bearings using a Vortex mixer. DNA was extracted from the bulked leaf samples with phenol-chloroform as described in our earlier papers (Shavrukov et al., 2016; Zotova et al., 2018). One microliter of DNA was checked on a 0.8% agarose gel to assess quality, and concentration was measured by Nano-Drop (Thermo Fisher Scientific, United States).

To identify SNPs in the GoI and compare them with annotated accessions in databases, primers were designed in exon regions flanking introns (Supplementary material 1). PCR was performed in 60 μl volume reactions containing 8 μl of template DNA adjusted to 20 ng/μl, and with the following components in the final concentrations listed: 1xPCR Buffer, 2.2 mM MgCl_2_, 0.2 mM each of dNTPs, 0.25 μM of each primer and 4.0 units of Taq-DNA polymerase in each reaction (Maxima, Thermo Fisher Scientific, United States). PCR was conducted on a SimpliAmp Thermal Cycler (Thermo Fisher Scientific, United States), using a program recommended by the Taq-polymerase manufacturer, with the following steps: initial denaturation, 95°C, 4 min; 35 cycles of 95°C for 20 s, 55°C for 20 s, 72°C for 1 min, and final extension, 72°C for 5 min. Single bands of the expected size were confirmed after visualization of 5 μl of the PCR product in 1% agarose gel. The remaining PCR reaction volume (55 μl) was purified using FavorPrep PCR Purification kit (Favorgene Biotec Corp., Taiwan) following the Manufacturer’s protocol. The concentrations of purified PCR products were measured using NanoDrop (Thermo Fisher Scientific, United States) and later used as a template (100 ng) in a sequencing reaction with the Beckman Coulter Sequencing kit, following the Manufacturer’s protocol. Sanger sequencing and analysis of results were performed on a Beckman Coulter Genetic Analysis System, Model CEQ 8000 (Beckman Coulter, United States) following the Manufacturer’s protocol and software at S. Seifullin Kazakh AgroTechnical University, Astana (Kazakhstan). The identified SNPs were used to design allele-specific primers that were applied in Amplifluor-like SNP analysis. Two fully sequenced chickpea accessions, ICC4958 of the Desi ecotype, and Frontier of the Kabuli ecotype, were used as the reference genomes^6^.

### SNP Amplifluor Analysis

Amplifluor-like SNP analysis was carried out using a QuantStudio-7 Real-Time PCR instrument (Thermo Fisher Scientific, United States) as described previously (Jatayev et al., 2017; Zotova et al., 2018) with the following modifications: Each reaction contained 3 μl of template DNA adjusted to 20 ng/μl, 5 μl of Hot-Start 2xBioMaster (MH020-400, Biolabmix, Novosibirsk, Russia^7^) with all other components as recommended by the manufacturers, including MgCl_2_ (2.0 mM). One microliter of a mixture of two fluorescently labeled Universal probes was added (0.25 μM each) and 1 μl of allele-specific primer mix (0.15 μM of each of two forward primers and 0.78 μM of the common reverse primer). Four microliter of Low ROX (Thermo Fisher Scientific, United States) was added as a passive reference label to the Master-mix as prescribed for the qPCR instrument. Assays were performed in 96-well microplates. Sequences of the Universal probes and primers as well as the sizes of amplicons are presented in Supplementary material 1.

PCR was conducted using a program adjusted from those published earlier (Jatayev et al., 2017; Zotova et al., 2018): initial denaturation, 95°C, 2 min; 14 “doubled” cycles of 95°C for 10 s, 60°C for 10 s, 72°C for 20 s, 95°C for 10 s, 55°C for 20 s and 72°C for 50 s; with recording of allele-specific fluorescence after each cycle. Genotyping by SNP calling was determined automatically by the instrument software, but each SNP result was also checked manually using amplification curves and final allele discrimination. Experiments were repeated twice over different days, where two technical replicates confirmed the confidence of SNP calls.

### Statistical Analysis

IBM SPSS Statistical software was applied to calculate means, standard errors, and to estimate the probabilities for significance using ANOVA tests.

## Results

### Bioinformatics and Comparative Phylogenetic Analysis

During the initial screening of SNP No. 2103, rs853191221 [*C. arietinum*] within the chickpea SNP database (Supplementary material 2), NCBI BLAST analysis revealed the closest nucleotide accession to be XM_012715175.1, encoding a Ras-related protein in *C. arietinum* with the corresponding *RabC1-*like gene (LOC101496214, transcript variant X2, mRNA). We designated this gene as the isoform *CaRabC-1a*.

To identify the full list of all members of *CaRab* genes in chickpea, bioinformatics approaches were used to search and analyze annotated sequences and whole genome sequences available in public databases using comparisons to the reference genome of *A. thaliana*. As a result, eight sub-families of *CaRab* gene were identified, with 54 isoforms. The corresponding accession IDs for the genes and proteins, as well as references to *Arabidopsis* genes with the highest level of similarity are shown in Table 2.

**Table 2:** The eight identified sub-families (*RabA* – *RabH*) of the chickpea *CaRab*, with 54 isoforms and their corresponding accession ID listed for genes and proteins as well as reference to closest genes in *Arabidopsis*.

Clades of *Rab* genes in plants	Group of *Rab* genes in mammals	Chromosome in chickpea	KEGG ID/NCBI Gene ID in chickpea	NCBI protein ID in chickpea	ID of closest gene in *Arabidopsis thaliana*
*RabA-1b*	*Rab11*	Ca1	101493366	XP_004489015	At1g16920
*RabA-1b^∗^*		Unknown	101507798	XP_004514628	At1g16920
*RabA-1c*		Ca6	101498968	XP_004505464	At5g45750
*RabA-1d*		Ca1	101504539	XP_004487583	At4g18800
*RabA-1f*		Ca6	101497770	XP_004505114	At5g60860
*RabA-1f^∗^*		Ca1	101489774	XP_004485855	At5g60860
*RabA-1g*		Ca1	101509653	XP_004487187	At3g15060
*RabA-1g^∗^*		Ca6	101492905	XP_004505524	At3g15060
*RabA-2a*		Ca1	101495904	XP_004485429	At1g09630
*RabA-2b*		Ca6	101503536	XP_004503210	At1g07410
*RabA-2b^∗^*		Ca2	101512788	XP_004490850	At1g07410
*RabA-2d*		Ca6	101512110	XP_004507156	At5g59150
*RabA-3*		Ca3	101490262	XP_004494844	At1g01200
*RabA-3^∗^*		Ca7	101511394	XP_004510635	At1g01200
*RabA-4a*		Ca5	101489316	XP_004502410	At5g65270
*RabA-4a^∗^*		Ca6	101515580	XP_004504100	At5g65270
*RabA-4c*		Unknown	101491858	XP_004516148	At5g47960
*RabA-4d*		Ca3	101508321	XP_004494010	At3g12160
*RabA-4d^∗^*		Ca4	101501691	XP_004497938	At3g12160
*RabA-5a*		Ca4	101498836	XP_004495529	At5g47520
*RabA-5b*		Ca3	101509902	XP_004492211	At3g07410
*RabA-5e*		Unknown	101508123	XP_004515580	At1g05810
*RabA-6a*		Ca8	101513235	XP_004512724	At1g73640
*RabA-6a^∗^*		Ca6	101500012	XP_004503285	At1g73640
*RabB-1b*	*Rab2*	Ca2	101515168	XP_004489550	At4g35860
*RabB-1c*		Ca1	101501307	XP_004486381	At4g17170
*RabB-1c^∗^*		Ca7	101496042	XP_004510833	At4g17170
***RabC-1a***	***Rab18***	**Ca4**	**101496214**	**XP_004498372**	**At1g43890**
***RabC-1b***		**Ca5**	**101488438**	**XP_004502943**	**At1g43890**
***RabC-1c***		**Ca6**	**101490080**	**XP_004503936**	**At1g43890**
***RabC-2a***		**Unknown**	**101497183**	**XP_004515929**	**At5g03530**
***RabC-2a^∗^***		**Ca4**	**101498490**	**XP_004496130**	**At5g03530**
*RabD-1*	*Rab1*	Ca1	101495577	XP_004485428	At3g11730
*RabD-2a*		Ca3	101506934	XP_004492924	At1g02130
*RabD-2a^∗^*		Unknown	101514122	XP_004515343	At1g02130
*RabD-2c*		Ca7	101496365	NP_001265926	At4g17530
*RabE-1a*	*Rab8*	Ca4	101497052	XP_004495000	At3g53610
*RabE-1a^∗^*		Ca1	101515594	XP_004487032	At5g59840
*RabE-1b*		Ca3	101504780	XP_004494002	At3g53610
*RabE-1c*		Ca4	101491866	XP_004497298	At3g46060
*RabE-1c^∗^*		Ca6	101506447	XP_004505885	At3g46060
*RabF-1*	*Rab5*	Ca4	101504907	XP_004496241	At3g54840
*RabF-2b*		Ca6	105851137	XP_012572119	At4g19640
*RabG-3a*	*Rab7*	Ca4	101504052	XP_004496410	At4g09720
*RabG-3b*		Ca2	101498677	XP_004489906	At1g22740
*RabG-3b^∗^*		Ca6	101514161	XP_004504012	At1g22740
*RabG-3c*		Ca6	101510738	XP_012573047	At3g16100
*RabG-3d*		Ca1	101509216	XP_004486740	At1g52280
*RabG-3e*		Ca6	101513957	XP_012573072	At1g49300
*RabG-3f*		Ca6	101496247	XP_004507422	At3g18820
*RabH-1d*	*Rab6*	Ca7	101507228	XP_004508989	At2g44610
*RabH-1d^∗^*		Ca8	101496604	XP_004511760	At2g22290
*RabH-1e*		Ca5	101492440	XP_004502420	At5g10260
*RabH-1e^∗^*		Ca6	101507522	XP_004504075	At5g10260

*The sub-family *CaRabC* studied in this paper is indicated in bold type.*

**FIGURE 1:**
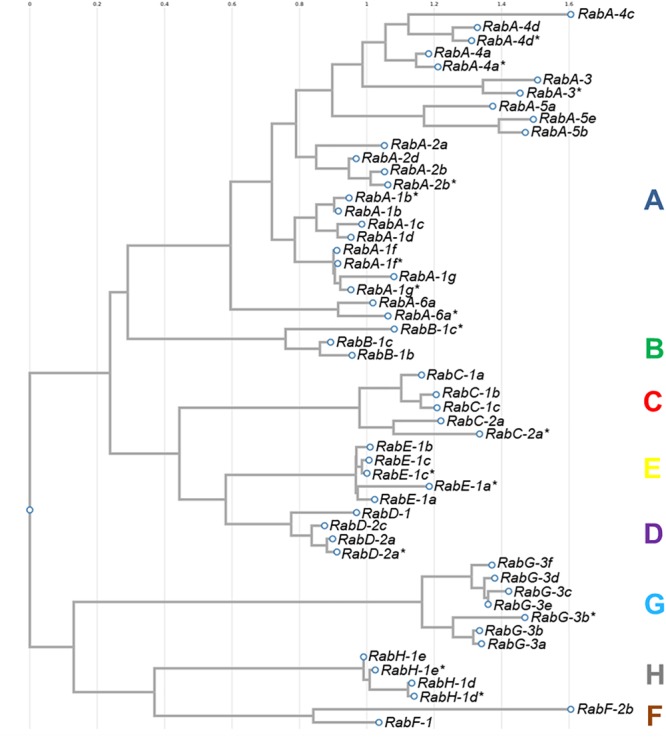
Phylogenetic tree of 54 *CaRab* gene isoforms identified in chickpea using the FastTree v2.1.8 program. Clades are designated by colored letters identical to the sub-family *CaRab* gene name.

The sequences of all 54 isoforms of *CaRab* genes identified in chickpea were used to construct a phylogenetic tree (Figure 1). Eight distinct clades were identified in the molecular dendrogram, and the letter corresponding to each sub-family name is used to distinguish the corresponding clade. The biggest and most diverse was Clade A, the *CaRabA* gene sub-family while Clades B and F contained only two accessions each. Clades D, G, H and F are molecularly similar, but most distanced from other sub-families of the *CaRab* gene. The sub-family *CaRabC* contained five isoforms with the closest sub-families being *CaRabD* and *CaRabE* (Figure 1).

**FIGURE 2:**
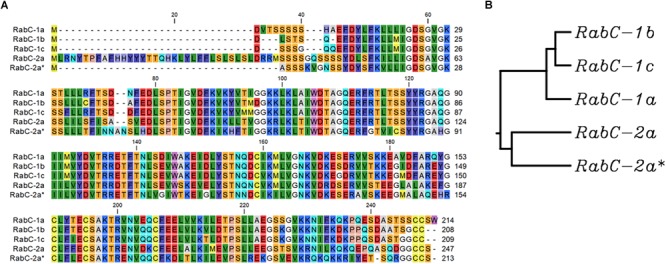
A comparison of amino acid sequences **(A)**, and Rooted UPGMA phylogenetic tree with branch length **(B)** of the five isoforms of CaRabC proteins identified in chickpea. Multiple sequence alignment conducted presented using CLC Main Workbench software.

Protein sequence analysis of five isoforms from sub-family *CaRabC* (Figure 2) showed distinct separation of CaRabC-1 from CaRabC-2. The closest molecular similarity was found between CaRabC-1b and CaRabC-1c with the next greatest similarity shared with CaRabC-1a, while CaRabC-2a and CaRabC-2a^∗^ were the most diverged from all others (Figure 2).

### RT-qPCR and Gene Expression Analysis

Primers for RT-qPCR analysis were designed based on the alignment and comparison of CDS sequences of five identified *CaRabC* isoforms listed in Table 2. To estimate the total expression level of all five *CaRabC* genes combined, common primers with degenerative nucleotides were designed based on the longest consensus regions in the alignments. In addition, 3’-ends of gene-specific primers were designed for specific SNPs to maximize the specificity of qPCR analysis for each of the five isoforms of *CaRabC* gene (Supplementary material 3).

**FIGURE 3:**
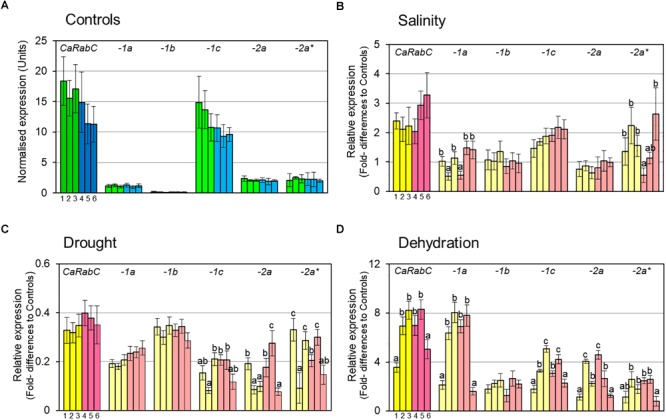
RT-qPCR analysis of *CaRabC* gene family expression in chickpea leaves: **(A)** In favorable, non-stressed conditions (Controls) for 3 Kabuli and 3 Desi (dark green and dark blue, respectively); and the relative gene expression compared to Controls under: **(B)** Gradual salt stress application, 150 mM NaCl, 7 days; **(C)** Slowly developing drought in pots with soil, 12 days; and **(D)** Rapid dehydration of detached leaves, 6 h, room temperature. All isoforms of *CaRabC* gene combined (darker colors) and the five separate isoforms (lighter colors) of the *CaRabC-1a, -1b, -1c, -2a* and *-2a^∗^* (for corresponding gene family) were analyzed separately. Each set contained six chickpea accessions, including three Kabuli ecotypes, shown in yellow (**1**, Yubileiny; **2**, ICC-7255; and **3**, ICC-4841), and three Desi ecotypes, shown in pink (**4**, ICC-1392; **5**, ICC-4918; and **6**, ICC-12726). Data were normalized using an average for two reference genes, calculated with ANOVA, and are presented as means for three biological and two technical replicates ± SE, shown as error bars. Significant differences (at least for *P* > 0.95) for each gene isoform and within each set of chickpea accessions are shown by different letters according to ANOVA tests.

Initially, the expression level of *CaRabC* gene was determined in control plants grown under favorable conditions for all isoforms combined, as well as for each of them separately (Figure 3A). All six studied chickpea accessions, 3 Kabuli and 3 Desi (dark green and dark blue, respectively, in Figure 3A), showed a very high level of total *CaRabC* gene expression, ranging from 11.2 to 18.4 relative expression units, with non-significant differences among the six studied genotypes. The expression level of a single isoform of *CaRabC-1c* had maximal (63–88%) contribution in the *CaRabC* gene expression in total. Two isoforms, *CaRabC-2a* and *-2a^∗^*, both showed very similar levels of 1.9–2.5 expression units. A level of around 1 expression unit was observed in the isoform *CaRabC-1a*, similar to the average level for the two reference genes used in this study. An extremely low level of expression (approximately 10-fold lower than both reference genes) was shown for the last isoform *CaRabC-1b* (Figure 3A).

For salinity stress (Figure 3B), a high level of expression of the total *CaRabC* gene family was observed with 2-3.3-fold higher expression relative to Controls, but no significant differences were found within each set of six studied accessions due to relatively wide variability between replicates. In all studied genotypes, the isoform *CaRabC-1c* made the highest contribution to the gene expression (around 1.5–2-fold above the Controls). Only two accessions, No. 2 (ICC-7255, Kabuli) and No. 6 (ICC-12726, Desi), showed a higher level of *CaRabC-2a^∗^* isoform expression (2.2- and 2.6-fold, respectively) but these data were quite variable. Significant genetic variation was found for expression levels of *CaRabC-1a* and *CaRabC-2a^∗^*. Expression levels of two isoforms, *CaRabC-1b* and *CaRabC-2a*, did not differ from Controls (Figure 3B).

A different expression pattern for the *CaRabC* gene family was found for the drought experiment, where total expression was down-regulated by 0.3–0.4-fold compared to Controls (Figure 3C). The highest contribution to gene expression was made by the isoform *CaRabC-1b*. There was no significant genetic variation for *CaRabC-1a* and *CaRabC-1b* among the studied germplasm while the other three isoforms were quite variable (Figure 3C).

In contrast, rapid dehydration of detached leaves resulted in an up-to 8-fold increase of expression for the total *CaRabC* gene family expression, as well as isoform *CaRabC-1a*, compared to controls (Figure 3D). With the exception of *CaRab1b*, significant genetic variation was observed among the studied chickpea accessions for all other isoform expression profiles.

### Amplicon Sequencing Showed an SNP in the Candidate Gene CaRabC-1a

The initial SNP discovered was annotated at position 516 from the start-codon in the identified CDS, LOC101496214, based on the reverse-compliment order in the SNP-containing fragment. The full nucleotide sequence of the accession and position of this initial SNP is presented in Supplementary material 2.

**FIGURE 4:**
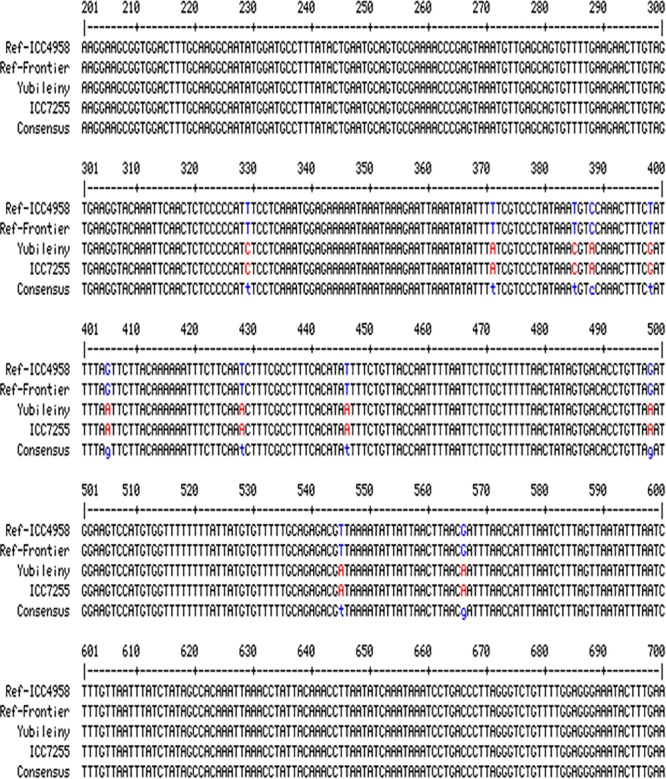
Partial alignments of *CaRabC-1a* (LOC101496214) amplicons. The alignment was produced with primers F5&R5 from the studied chickpea germplasm, Yubileiny and ICC7255, compared to two fully sequenced reference accessions, ICC4958 and Frontier. The studied amplicons were located on Chromosome 4, position 27,819,854–27,818,706 (Reverse order), in the reference accession ICC4958 (Desi ecotype), while the amplicon position in the second reference accession Frontier (Kabuli ecotype), was at 38,617,439–38,616,292 (Reverse order), also on Chromosome 4. Eleven identified SNPs are shown in red for the two studied accessions against those indicated in blue for the two reference accessions.

To check for the presence/absence of the initial SNP in the studied chickpea accessions, several pairs of primers were designed flanking the SNP. The most successful primer pair, F5&R5, amplified a fragment of 1148 bp. A fragment of the alignment showing polymorphic amplicons from the germplasm sequences compared to two fully sequenced reference chickpea accessions (ICC4958, Desi ecotype and Frontier, Kabuli ecotype) in *CaRabC-1a* is presented in Figure 4. The sequencing of the amplified fragments revealed the presence of 11 new SNPs in two chickpea accessions, Yubileiny and ICC7255, both Kabuli ecotypes (Table 1), compared to the two reference accessions. All 11 identified SNPs recorded high scores, and clear nucleotide peaks at the SNP positions were assessed manually. Interestingly, the initial SNP recorded in the database was monomorphic among the two reference accessions and two genotypes sequenced in our study.

### SNP Screening in CaRabC-1a Using Amplifluor-Like Markers

**FIGURE 5:**
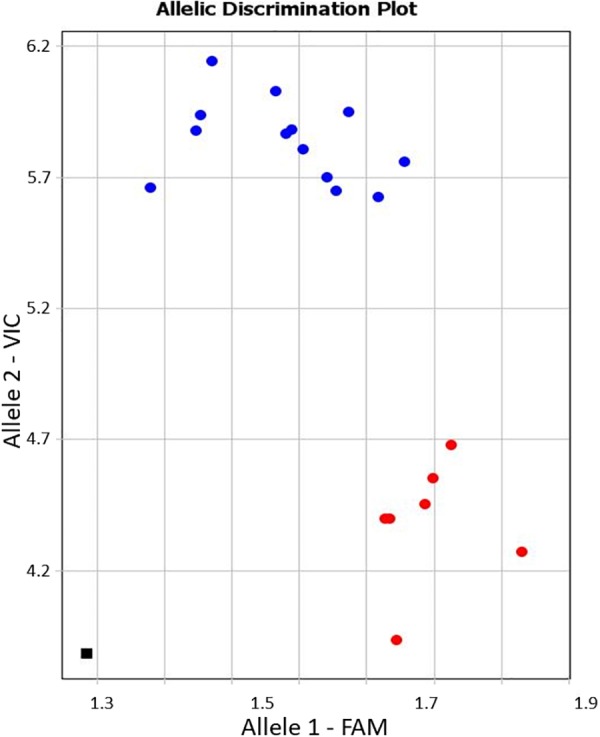
An example of allele discrimination in the chickpea germplasm collection using the Amplifluor-like SNP marker KATU-C22. *X*- and *Y*-axes show Relative amplification units, ΔRn, for FAM and VIC fluorescence signals, respectively. Red dots represent homozygote (*aa*) genotypes with allele 1 (FAM), and blue dots represent homozygote (*bb*) genotypes for allele 2 (VIC) identified with automatic SNP calling. The black square shows the no template control (NTC) using water instead of template DNA.

Allele-specific primers, KATU-C22-F&R, were designed for one of the selected SNPs from the 11 identified in the studied fragment of isoform *CaRabC-1a* to use with Amplifluor-like genotyping analysis. Details on the design of primers and positions of the studied SNPs are presented in Supplementary material 4. The example in Figure 5 shows allele discrimination using Amplifluor-like SNP marker KATU-C22, where allele 1 (FAM) has been identified in chickpea accessions with SNP genotypes similar to reference accessions ICC4958 and Frontier but allele 2 (VIC) was found in germplasm similar to Yubileiny and ICC7255 (Figure 5).

## Discussion

Rab-GTP proteins are well known in oncology studies in human and animals, but in plants there is increasing evidence that they play a central role in the tolerance to abiotic and biotic stresses. Nevertheless, it appears that the mechanism of membrane trafficking with which they are associated is similar in cells of both humans and plants. Most *Rab* genes of the eight clades represented in the molecular phylogenetic tree in plants, have similar corresponding groups of genes in human and other animal genomes. A greater or lesser diversity of isoforms for each clade of *Rab* genes just reflects the differing outcomes of evolution in the plant and animal kingdoms.

In plants, the most studied groups of *Rab* genes are from Clades G and H, where multiple vacuolar trafficking pathway components were demonstrated (Vernoud et al., 2003; Peng et al., 2014; Uemura and Ueda, 2014; Brillada and Rojas-Pierce, 2017). These types of *Rab* genes encode proteins that have been associated with a response to salinity and osmotic stresses, and are thought to associate with pre-vacuolar vesicles. Thus, Rab proteins may enhance relocation of Na^+^ ions to the vacuole, after they reach a toxic level in the cytoplasm of cells. Whilst there has been less attention placed on other groups of *Rab* genes, including the diverse Clade A with its many isoforms and the non-diverse Clade B with only two gene members, there is practically nothing known about Clade C of *Rab* in plants (Vernoud et al., 2003; Jha et al., 2014; Rehman and Sansebastiano, 2014; Lawson et al., 2018). Despite the strong similarity between *A. thaliana* and *C. arietinum*, our bioinformatic results show significant differences in the number of *Rab* isoforms in most clades.

In the work described here, 54 isoforms of *CaRab* genes were identified in chickpea, indicating an evolutionary reorganization when compared to *A. thaliana*, where 57 *AtRab* isoforms have been identified (Vernoud et al., 2003). Clade C in the chickpea dendrogram has not been previously identified, described or studied, and contains the five isoforms: *CaRabC-1a, -1b, -1c, -2a* and *-2a^∗^*. The first three isoforms show similarity to *AtRabC-1* (At1g43890, Table 1) while the latter two isoforms in chickpea were similar to another single isoform *AtRabC-2a* (At5g03530). The isoform *AtRabC-2b* (At3g09910), listed in a comprehensive analysis of the *Rab* genes in *A. thaliana* (Vernoud et al., 2003), has no ortholog in the *C. arietinum* genome. To avoid any misunderstanding with the classification of *CaRabC-2a* and *-2a^∗^* isoforms, we have used an asterisk instead of another letter, to indicate its very similar polypeptide structure.

Following the bioinformatics study, the expression analyses of total *CaRabC* for all five isoforms revealed high levels of expression of the gene family in leaves of non-stressed young chickpea plants compared to two reference genes (Figure 3A). More importantly, a single isoform, *CaRabC-1c*, made the major contribution to the gene expression, indicating a very active role of this isoform in chickpea plant development under non-stressed conditions. In the absence of other reports comparing expression of individual and combined (bulk) isoforms of *Rab* genes in plants, our conclusions await further verification and discussion.

Under salt stress, the dominance of the *CaRabC-1c* isoform in expression profiles was not as pronounced as under control conditions and was more comparable to other isoforms in some of the studied chickpea accessions, particularly *CaRabC-1a* and *CaRabC-2a^∗^*. Therefore, at least three isoforms of *CaRabC* were salinity-responsive and the two latter ones were strongly genotype-dependent (Figure 3B).

An unexpected result was found in the comparison of *CaRabC* gene expression in response to slowly progressing drought of whole plants and rapid dehydration of detached leaves. Only a few reports have described expression of different genes in parallel experiments with drought and dehydration. For example, a peroxisomal isoform of *APX*, Ascorbate peroxidase, was down-regulated under strong drought but up-regulated in desiccated leaves in a cultivar of cowpea, *Vigna unguiculata* (D’Arcy-Lameta et al., 2006). Similar results were reported for two genes associated with loss of water during slow drought progression compared to rapid dehydration of barley leaves: *HvMT2*, a metallothionein-like protein, and *2HvLHCB*, Chlorophyll *a-b* binding protein of LHCII type III (Gürel et al., 2016). Therefore, there are examples of genes related to drought and dehydration that can be down- and up-regulated, in several plant species. However, our results show for the first time that all isoforms of *CaRabC* were strongly down-regulated under the slowly developing drought, but very strongly up-regulated in rapidly dehydrated leaves (Figure 3C,D).

Amplifluor-like SNP markers and other molecular markers are very helpful in identifying genetic polymorphisms in diverse germplasm accessions. In the current study, the molecular marker KATU-C22 was useful for genotyping one isoform *CaRabC-1a* (Figure 5). This allows for tracking of the different variants of this gene and the possibility of linking variants with an associated phenotype. Additional markers are now needed for all other isoforms of *CaRabC* and other GoI, but this will require further investment in sequencing in the future. It also may be worth looking for SNPs in the upstream promoter regions of the gene family, since this could explain the variation in expression between the genotypes.

*CaRabC* is just one sub-family from a large *CaRab* gene family involved in controlling cell membrane trafficking, and like the other *Rab* genes investigated to date (reviewed in Flowers et al., 2018), it is responsive and potentially associated with the adaptation of plants to abiotic stresses. For comparison, in the bacteria *Salmonella*, the Rab18 protein (related to RabC in plants) is actively involved in endocytosis and is localized in the early endocytic compartment of cells (Hashim et al., 2000). In plants, there is increasing evidence for the role of endocytosis under salinity and osmotic stress (Martín-Davison et al., 2017). The implications of increased endocytosis during these stresses would be a reduction in total plasma-membrane area, thereby limiting water loss from the cell through a decrease in the number of aquaporins. Additionally, it may represent a mechanism to obtain Na^+^ ions directly from outside the cell for accumulation in the vacuole, thus keeping the cytoplasmic level of Na^+^ low (Baral et al., 2015). In future work, we hope to explore the role of *CaRabC* on endocytosis and Na^+^ compartmentalization. There has been very little work published to date concerning endocytosis and extended drought. The different responses shown in the changes in expression observed in this study between salinity and dehydration (both components of osmotic stress), is intriguing and probably indicative of the underlying biological role of RabC proteins themselves.

Further research is required in several selected chickpea accessions to assess tolerance to salinity, drought and rapid dehydration. This would allow us to explore possible associations between sequence variants and levels of stress tolerance. The genotype-dependent role of each isoform of *CaRabC* as well as other genes from the gene family will be studied, and we plan to carry out these experiments in the near future. These new experiments should elaborate on the mechanism and clarify the suggested roles of these proteins in cell polarization and recycling to the plasma membrane, as suggested by Vernoud et al. (2003) and Rutherford and Moore (2002), respectively. Hopefully, our study of *CaRabC* extends the knowledge of *Rab* gene family structure and function in plants.

## Author Contributions

GK conducted the experiments with chickpea germplasm and the genotyping with Amplifluor-like SNP analysis. AK and SJ supervised the experiments and interpreted the results. AsZ conducted the experiments with plant stresses and sampling. AyZ carried out sequencing. AT worked with plants in the field trial. BA coordinated the experiments in the field. SL analyzed gene sequences in databases and wrote the corresponding sections. CS analyzed the results, and revised and edited the manuscript. CJ analyzed the qRT-PCR data and revised the corresponding section. KS coordinated the qRT-PCR study and revised other sections. PL supervised the study and revised the final version of the manuscript. YS coordinated all experiments and wrote the first version of the manuscript.

## Conflict of Interest Statement

The authors declare that the research was conducted in the absence of any commercial or financial relationships that could be construed as a potential conflict of interest.

## Footnotes

1 https://www.ncbi.nlm.nih.gov/snp

2 https://www.genome.jp/tools/blast

3 https://legumeinfo.org/organism/Cicer/arietinum

4 https://www.genome.jp/tools-bin/clustalw

5 https://www.qiagenbioinformatics.com/products/clc-main-workbench

6 http://www.cicer.info/databases.php

7 http://biolabmix.ru/en/products
