# Supplementary material for: Intracellular Vesicle Trafficking Genes, RabC-GTP, Are Highly Expressed Under Salinity and Rapid Dehydration but Down-Regulated by Drought in Leaves of Chickpea (Cicer arietinum L.)
Source: Front Genet. 2019 Feb 7;10:40. doi: 10.3389/fgene.2019.00040 (PMC6374294; doi:10.3389/fgene.2019.00040)
Supplement: Supplementary file 1 [file Data_Sheet_1.PDF]

Supplementary material 1

Primers designed and used for Amplifluor-like SNP analysis

Forward primer for Frontier/ ICC4958-type allele:

KATU-C22-SNP-F7: GAAGGTGACCAAGTTCATGCTCCCTATAAA**T**GT**C**CAAACTTTC**T**

Forward primer for Yubileiniy/ICC7255-type allele:

KATU-C22-SNP-F8: GAAGGTCGGAGTCAACGGATTCCCTATAAA**C**GT**A**CAAACTTTC**G**

Common Reverse primer

KATU-C22-SNP-R2: AGGTGTCACTATAGTTAAAAAGC

Reverse compliment GCTTTTTAACTATAGTGACACCT

Size of amplicon: 119 bp

Sequence of two Universal probes

Universal probe 1:

5′-FAM-AGCGATGCGTTCGAGCATCGC(T*-BHQ1)*GAAGGTGACCAAGTTCATGCT*-3′

Universal probe 2:

5′-VIC-AGGACGCTGAGATGCGTCC(T*-BHQ1)*GAAGGTCGGAGTCAACGGATT*-3′

Primers designed and used for sequencing

KATU-C22-F5: CTCAACAAATCAAGACTGTATCAAG

KATU-C22-R4: CACCATGAGCAACAGCTACTAG

KATU-C22-R4 (Rev-Com): CTAGTAGCTGTTGCTCATGGTGA

Size of amplicon: 1,235 bp

Sequence of Reference genes used for qPCR

CaCAC-F: CATGGACTAGACCACCAATTCA

CaCAC-R: AACAGTGTTGTACCCGCTCTTT

Size of amplicon: 110 bp (Reddy et al., 2016)

CaGAPDH-F: CCAAGGTCAAGATCGGAATCA

CaGAPDH-R: CAAAGCCACTCTAGCAACCAAA

Size of amplicon: 65 bp (Garg et al., 2010)

Supplementary material 2

Selected annotated SNP in chickpea database.

2103. rs853191221 [*Cicer arietinum*]
GAATTTGTACCTTCACTACAAGTTC[G/T]TCAAAACACTGCTCAACATTTACTC

Chromosome: Ca4:38617150 Gene:LOC101496214 (GeneView) Functional Consequence: missense Validated: no info

Annotated mRNA sequence from NCBI database, closest to the above fragment with SNP (with reverse-compliment orientation). Start- and Stop-codons are indicated in green and red, respectively. The position of annotated SNP is indicated in yellow.

>XM_012715175.1 PREDICTED: Cicer arietinum ras-related protein RABC1-like (LOC101496214), transcript variant X2, mRNA

```
ATAGTAATTTCAGAAAACCAAACGGTTCAGCGGCGCTAGCATACGCCAAGTTTTCTTTGACTTTCCGCAA
CCTTCTACTAAATACTTTACTTCTTAGCTTTTTCTTTTCCTTCTGTTTCCACATTCCACAGAATTGATTC
AACCCAAGTCTTGATTTTCCAAGGAACCGAAGAAAATATCTCTGAGATCCAATCTAAACAAAACAATATC
TGAATGGATGTTACTTCCTCCTCGTCGAGTCACGCCGAATTCGATTACTTATTCAAGCTTCTGTTAATTG
GTGATTCTGGAGTCGGCAAAAGCACGCTGCTTCTCAGATTCACCTCTGATAATTTCGAGGATCTTTCTCC
TACCATCGGTGTAGACTTCAAAGTGAAATATGTTACAATTGGTGGAAAAAAGTTAAAACTTGCTATTTGG
GACACAGCTGGACAAGAAAGGTTTAGAACACTTACCAGTTCATATTATAGAGGTGCACAAGGAATAATTA
TGGTGTACGATGTAACACGGCGGGAAACTTTTACAAATCTATCTGATATATGGGCTAAAGAAATTGACTT
ATACTCAACAAATCAAGACTGTATCAAGATGCTTGTAGGAAACAAAGTTGACAAGGAAAGTGAAAGGGTT
GTTAGCAAAAAGGAAGCGGTGGACTTTGCAAGGCAATATGGATGCCTTTATACTGAATGCAGTGCGAAAA
CCCGAGTAAATGTTGAGCAGTGTTTTGAAGAACTTGTAGTGAAGATTTTGGAGACACCAAGCCTCTTGGC
TGAGGGCTCATCTGGTGTGAAAAAAAACATCTTCAAGCAGAAACCACAAGAGTCTGATGCATCAACTAGT
AGCTGTTGCTCATGGTGATTATGATGTATTTAAAGAGCTATCACCTTCCACTTTTCAGTATGATGTTAAG
ACATTTCATTACATGTACCAAAAAACTAATTTCAATTTGTTGCGATCAATCTTTAAAATTTGCCTACTTT
TAATGGTCTCTTTCATTTTA
```

Supplementary material 3

Alignment of five isoforms of the *CaRabC* gene identified in chickpea. Green boxes in the consensus sequence indicate the binding sites of qPCR primers for expression analysis of *CaRabC* gene for all five isoforms combined. The positions of qPCR primer pairs for expression analysis of *CaRabC* gene targeting each isoform specifically are shown in grey boxes.

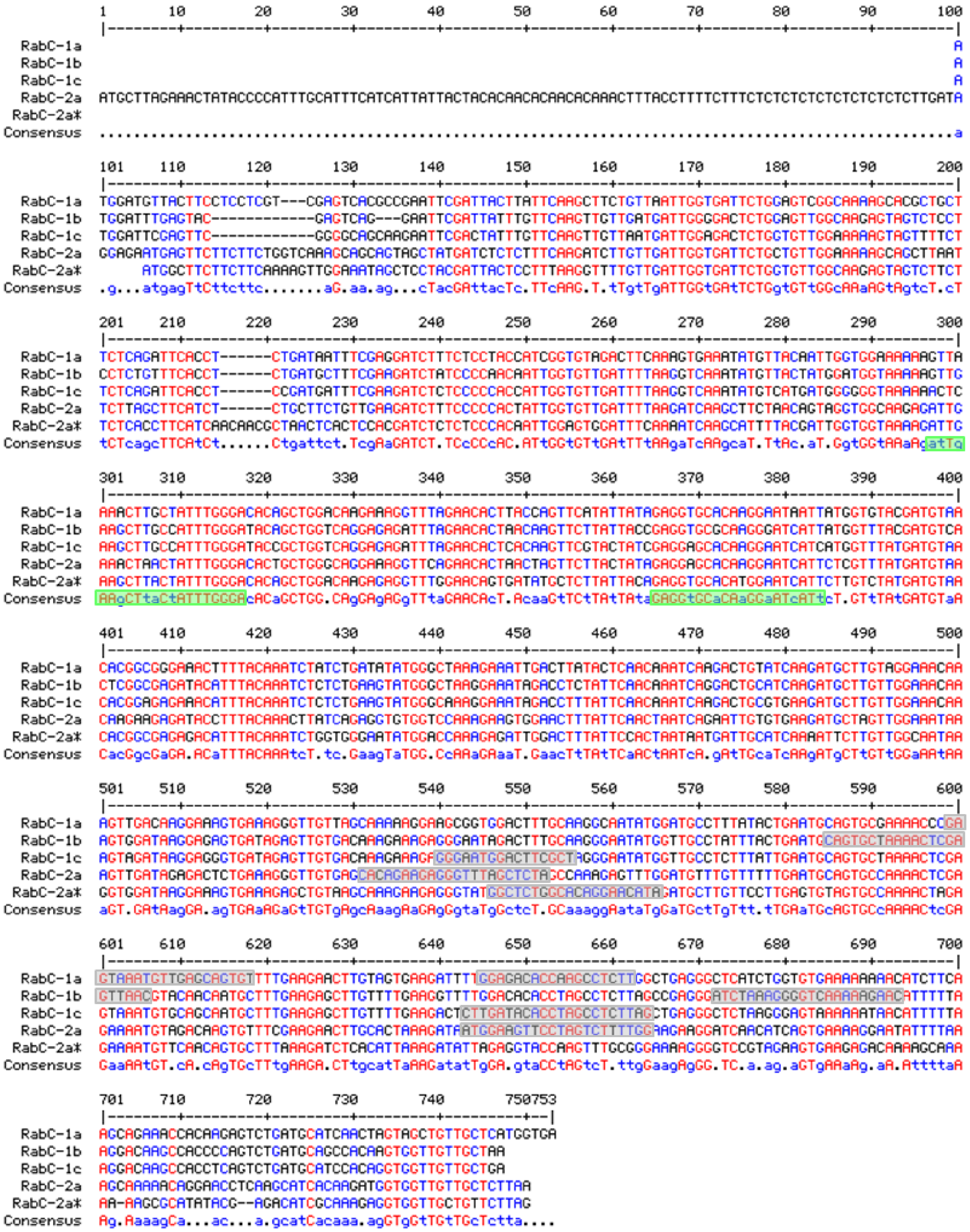

Supplementary material 4

The fragment of genomic DNA, chromosome 4, retrieved from chickpea database matching the sequence LOC101496214 from the Supplementary 2 above. Start- and Stop-codons are indicated in green and in red, respectively. Exons are shown in Capital letters and introns are by letters in normal case. Primers used for sequencing are highlighted in pink. Primers used for Amplifluor-like SNP analysis (as shown in the Supplementary material 1) are highlighted in blue. Annotated and used SNPs are shown in yellow, in Capital and in normal case letters, respectively. The identified 11 SNP are shown in Bold in squired brackets.

>Ca4: 38,622,857 - 38,615,990 (reverse-complement)

```
ATAGTAATTTCAGAAAACCAAACGGTTCAGCGGCGCTAGCATACGCCAAGTTTTCTTTGA
CTTTCCGCAACCTTCTACTAAATACTTTACTTCTTAGCTTTTTCTTTTCCTTCTGTTTCC
ACATTCCACAGAATTGATTCAACCCAAGTCTTGATTTTCCAAGGAACCGAAGAAAATATC
TCTGAGATCCAATCTAAACAAAACAATATCTGAATGGATGTTACTTCCTCCTCGTCGAGT
CACGCCGAATTCGATTACTTATTCAAGCTTCTGTTAATTGGTGATTCTGGAGTCGGCAAA
AGCACGCTGCTTCTCAGATTCACCTCTGATAATTTCGAGGATCTTTCTCCTACCATCggt
tacttcactttactccctaaactaattcctcatttccattttcattttgcctttttttat
ttattttatttctatacccaattacacttttcacgtcttaattagcgtgagtgcgtgcgt
ttattgtaattctcaacagttgtttgtagtaaattttcatttgtttttaaaaatctaaaa
aaattaacatttaaattatgaaatactaaaataaaaagacttaaaaaatgatataacatt
cttaataagttaaataacttttttttttttactacaaattaaaaaacataaaaacatgaac
tttccctctaagaacggagagggaaaaaaaagagcgagagtgagatgaagacggaggcgc
ataatctactgcagaagctccatcgaagaaccgagaagtgtagcgtggggtgtcccatac
tagatcgctgtcttaacggcggtataccatgcaaatcagtaacggaactcgtcgccgaaa
gcggaagcggcaaaacccaaatctgtctccaactcgtcctctccgctcagcttccaccct
cccacggcggcctatcatcatcatcactctacatacacaccgaattccctttcccatcc
gccgcctaaaacaactctcccgcattttcatttcatctcattccgaacttctcggctcct
ccgatccctgtgaccgtgttttcctccgtggcgttcattcagccgacgaattcgtcgatg
caatgccggaaatcgagtttttccttacttactggaaggctagattgttgcctgtgagag
tgattgttgttgattctattgctgctttgtttaggtctgattttgagaactctagtttgg
atcttagaaaaaggtcgtcgctgtttttcaagatttcgggtggattgaagtcgttggcgg
agaggtttgggttggctgtgctggttactaatcaggttgttgattttattggtgaggatg
aaggtttgaataatggaatgaggattgggaattttagtgagttgtattcctcgggtcgaa
gggtttgtcctgcgttaggtctttcatgggcaaattgtgttaactctaggttctttttat
ccatggatgacgaatatgaggagggtggtggtagggtaactaaaatgagaagacttagtg
ttgtttttgctcctcatttgcctcaatgttcttctcggtttgtcatcaccacagaaggtg
ttttcggtgtggagatgcataaaggaaatgtatgaattattgttattatgtatttatttc
ctgatattttcaagagggaaatatatgtatgtattaccatggtaagtgctgtacagaaat
taaaatagaatcaatggaagaggagaattggtttttgagcaacatcagttgtagacatgg
taatggagaagatattgaattttggagacataaatggatcgagtccaaacctttgatgga
ggaatttcccaagttgtactccttgtatcattaaaaatggtagtgtggccttattgggac
attagaattcagaacagttgacttggggtcttggaggtggaattctatattatgctcaag
agatacaattgcggatgttggaatcaattctatcggaggtcagaccagatttaattgatt
gagatagctggagttggtctctggaaagcaccactggtttttctgttaactcgtgttata
aggtattactctccaaagttctggttgctgaaactgataacaccagtttgcgagcgttga
aaatttgtggcagaatgatgtcctataaaaaattcatattttcggacagaggttgctgc
ccttgagacttccaactcgcgatgagttggttaaacaagcagttttattagatagccata
gctctgcttgtgtgctttgttttcaacaggaagaaacctgccagcacctgttttactcgt
gtggcagcatattgttagtctggaaggagattttagaatggatgggtgcctccttgggta
gtgaaagttgtggtgcccaacacttttttagaattcagtaatttgtttgtagggaggaaaa
acaagaaggttattaacttgatgtgggttgctactccttggtgctcttggagaatgataa
atgacattttatttaatggaggggtacttgatttgtccaagttggtcaacaatattaaga
cattatcttggatatggtttattaatagagcagtaaaaaacactccttatgtattttcca
ttaggttttcgacccccattgcttgtttgacttgtaggtaatacctatattgtaatgggt
tagagtaccccttatactctttttaatgccattttgcttattaaaaaaaccacgtgccc
tctcatgtttgtgtttttttattttatttttaatttactaatatccactctggacatgtg
agaaggatgtcaaaattgcgcaattgaaaaattagggggtcaaaagtgcaattaagtctt
```

tccttttatgaaaaaatttactttcccgtttctttttattagaaaaaattagttctactt
attagttgttttggaatttctagttacattaagcattctcccggcctcatttataaaaaa
attcaggaatttgttggacagacttgaataaatggatttaattatcattcttaatagtgg
ttaaaatgtaaatggtgattgggtgggtcaataaaatgtaaaggtatatttgaaattgtt
tattaataaaatgtatttattggttttgcaaacaatcttataaaaccggtatatagaaac
cgaggaaatattattcctgcaatttggttttaagattggatgagatgttcgtggtttctc
aatgtagttgtatttgaggaatgtcgatgtagatgatttttatttttttcctttttttaga
acacttgtctgagttgtctgacacgtattattagggttttgtacgagtgacaatgtctat
tgcaatatctgtcagacacgtctaaactcagtagtgtagctgcttcatggttaaggactt
tttgttcttgttcacagaaggacccttgagacgtgtagtgacttttgaggcttgaccagc
aggtttcatttggttcagggtattcatatatgatcaaactagtaacattcaagattgcag
ggatgtagg**c**aaaaagtgggatccaaattcactgttgtactgtaatgtaactacataaag
tagtatataatcttgatcattaatttgaaatcagacagtttggattacacattcactaaa
tcaactttaaataatttatgccactgatttatattagccgatcgaaatttgtaactacgt
cacttagtttagggaatctgatcttttgaaaaagtgatgtgaaattccactatgcgtcat
tagtaattttctagtatttcttgttgtatcacatgtacttctttttacctagtgtactct
ttcattatttatatgtaatgatttcaagctctctcatttttgtcaaaaaaaaaaaagcttc
tctctcatagtatgtgttaggaaaaattaaagaatcagaatagtggttgttgaatcatta
tccatcttatacctataaatgtaaacaaaatttatatttcattggggaaggccttgataa
cacctttccttggtgtaagaagtcatatgaaatgtgatggtttatgtgtggttcatgata
ctctttttatagtacctgtcactcaattataggttaaagtataactttcttcatttgttt
gcattgactcttttaaacttattacataatgcaGGTGTAGACTTCAAAGTGAAATATGTT
ACAATTGGTGGAAAAAAGTTAAAACTTGCTATTTGGGACAcaggtaatgtgacatatgct
gatttattttattggaaagtcaaaattctgttatttttccgtcattatgggattcttttc
tattctgcattcttcatattcatctaatgttttcaaaattctatagtatttaaggtgtgg
aacttcttcctattattataaacctgtatgctgcatattgagtgtggaagtgctagagct
ctcatttacttcactgttttgtgatataattgcaatgtatgccataccaacttaaatttt
caaaatttagtttttggattttctctgtcggcgattatcgattaacatatctttggatga
tgtctaagatttaacaacagaatttagcaaactccacaatgttgtttaaaatcagaatat
gcaaaatagactatatattcatgtcaaaggactgagtaaactttagtttgcacttagtgt
cattatcggaatgtttatacatttatattgtgttgagttcaacagaagattgaaagtttc
ctgtgtaaatttaacaaaacatattgcatagtatgatgttttagttgctatgtgctgtat
aaagcatttcttatagacagagatgaacaacttgtcttggtataataattcacttctctt
ttttacaaatttacatgactaaaattttgaagaagtttgttcaaccatatctgcaataag
ttagttacaaattttgtattttctcttcgtgCAGCTGGACAAGAAAGGTTTAGAACACTT
ACCAGTTCATATTATAGAGGTGCACAAGGAATAATTATGGgtatgctccttgatttactc
catttgttagctatgtttcctgcttgctatcccatgaatatgatgatgtactttgttttt
cttcttaatttgtatgaattctatcatgtttttgagacttaccggttcatattatagagg
tgcacaaggaataattatgggtatgctccttgatttactcaatttgttagctatgctcct
tgatttactccatttgttggctatatttcctgcttgctatcccatgaatatgatgatgta
ctttgtttttcttcctaatttgtttgaattttatcatgtgtttgagctgtaaatcatgca
acatggctatgcgtatagttcataatttatattataaacctatcattgaatctgggttat
gaacacagTGTACGATGTAACACGGCGGGAAACTTTTACAAATCTATCTGATATATGGGC
TAAAGAAATTGACTTATACTCAACAAATCAAGACTGTATCAAGATGCTTGTAGGAAACAA
AGTTGACAaggtacaccgtttacgaatttgcatgtgcaataccttcactcgaccaaaaat
ttgtgctttaagttttaattcactgattcagtaaaaaattgcttgtgaatatagtttgct
tattcatattttAGGAAAGTGAAAGGGTTGTTAGCAAAAAGGAAGCGGTGGACTTTGCAA
GGCAATATGGATGCCTTTATACTGAATGCAGTGCGAAAACCCGAGTAAATGTTGAGCAGT
GTTTTGA[A/C]GAACTTGTAGTGAaggtacaaattcaactctcccccat**[t/c]**tcctcaaatggag
aaaaataaataaagaattaaatatattt**[t/a]**tcgtccctataaa**[t/c]**gt**[c/a]**caaactttc**[t/g]**attt
ta**[g/a]**ttcttacaaaaaatttcttcaa**[t/a]**ctttcgcctttcacata**[t/a]**tttctgttaccaattt
taattcttgcttttttaactatagtgacacctgtta**[g/a]**atggaagtccatgtggttttttt
attatgtgtttttgcagagacg**[t/a]**taaaatattattaacttaac**[g/a]**atttaaccatttaatc
tttagttaatatttaatctttgttaatttatctatagccacaaattaaacctattacaaa
ccttaatatcaaataaatcctgacccttagggtctgttttggagggaaatactttgaagg
gctaatcctatattttaatatatatttgatattttaaatttttttgaaagagcaaacatg
ataaatgagcatataatatcatgttcttttaaaatcatgttcttttaatttaaaaaaaaa
atatcatataatatcatgttcttttaatttaaaaaaaaaatatcatataatatcatgttc
ttttaattttttttaaaaatcaaatatatataaaaatataaatttaaccctccaaagcc
ttctcctccaaaacccccaaaaaccaaactaacttgaagcaaaagataagattaaaatt
ggttttgctacccctatccaagcaccatttttcttttatttatttgcattaagtacaatt

cagagcaccttgagaaaatgaaagtactgattcaattttggattccttttgttatttccA
GATTTTGGAGACACCAAGCCTCTTGGCTGAGGGCTCATCTGGTGTGAAAAAAAACATCTT
CAAGCAGAAACCACAAGAGTCTGATGCATCAACTAGTAGCTGTTGCTCATGGTGATTATG
ATGTATTTAAAGAGCTATCACCTTCCACTTTTCAGTATGATGTTAAGACATTTCATTACA
TGTACCAAAAAACTAATTTCAATTTGTTGCGATCAATCTTTAAAATTTGCCTACTTTTAA
TGGTCTCTTTCATTTTATGCTTTGATGGGTGAGATTTACATAGTAGAAGAAATTTAAAAT
GAAAGATGTCTCTAAAGAATCAGAACAA
